# Cq values as an indicator for COVID‐19 outcomes: A study of the correlation between laboratory parameters

**DOI:** 10.1002/iid3.1326

**Published:** 2024-06-24

**Authors:** Hadi Safdari, Saeede Bagheri, Nasrin Talkhi, Elahe Saberi Teymourian, Mahdi Hosseini Bafghi, Mohammad Hossein Ahmadi

**Affiliations:** ^1^ Department of Laboratory Sciences, School of Paramedical Sciences, Faculty of Paramedical and Rehabilitation Sciences Mashhad University of Medical Sciences Mashhad Iran; ^2^ Department of Biostatistics, School of Allied Medical Sciences Shahid Beheshti University of Medical Sciences Tehran Iran

**Keywords:** COVID‐19, Cq level, laboratory parameters

## Abstract

**Objective:**

The ongoing outbreak of the respiratory disease coronavirus disease 2019 (COVID‐19) is currently presenting a major global health threat. This pandemic is unprecedented in recent human history. The objective of this study was to examine the relationship between cycle quantitation (Cq) and laboratory parameters in COVID‐19 patients, aiming to determine if Cq levels can provide valuable insights into the COVID‐19 disease.

**Methods:**

This study involved 234 participants who were divided into case and control groups. Real‐time PCR tests were used to diagnose COVID‐19 cases in the study participants. Blood tests, including complete blood count, C‐reactive protein (CRP), erythrocyte sedimentation rate (ESR), lactate dehydrogenase (LDH), D‐dimer, IgG, and IgM, were also conducted. Statistical analysis was performed using SPSS 22 software.

**Results:**

The findings showed that COVID‐19‐positive cases had significantly higher levels of the neutrophil‐to‐lymphocyte ratio (NLR), platelet‐to‐lymphocyte ratio (PLR), D‐dimer, ESR, CRP, and LDH compared to normal cases. Additionally, the case group had significantly lower lymphocyte and platelet counts. There was a statistically significant positive correlation between Cq levels and lymphocyte count (*r* = .124, *p* = .014). Conversely, there was a statistically significant inverse correlation between Cq levels and NLR (*r* = −.208, *p* = .017). Furthermore, the evaluation of hematological, inflammatory, and biochemical indexes in COVID‐19 patients using the receiver‐operating characteristics curve demonstrated statistically appropriate sensitivity and specificity.

**Conclusion:**

Our outcomes indicated a significant association between Cq levels and PLR, NLR, D‐dimer, CRP, and ESR in COVID‐19 patients. Consequently, including the report of laboratory parameters alongside Cq values offers a promising prognosis.

## Graphical abstract

This study demonstrated the correlation between laboratory parameters and Cq values as an indicator for the outcomes of coronavirus disease 2019 patients.

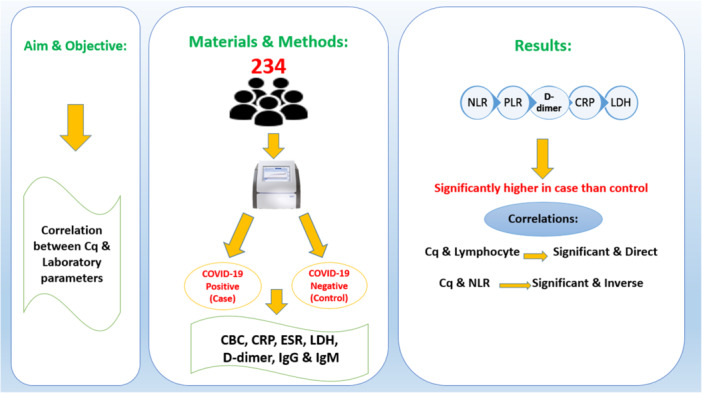

## 1 INTRODUCTION

The recent outbreak of the novel coronavirus disease (COVID‐19), caused by the severe acute respiratory syndrome coronavirus 2 (SARS‐CoV‐2), has witnessed a rapid increase in the number of infected patients worldwide.^1^ COVID‐19, first identified in Wuhan, China, in late December 2019, spread to 213 countries in 2020 and subsequently became a global pandemic.^2^ As of January 2023, approximately 663 million individuals have been infected with the coronavirus, resulting in 6.7 million deaths.^3^

Common symptoms of COVID‐19 include fever, cough, fatigue, shortness of breath, muscle pain, chills, sore throat, headache, and chest pain. However, these symptoms are nonspecific and can also be observed in other infections, such as influenza. Therefore, specific diagnostic tests for COVID‐19 are necessary to confirm suspected cases.^4^ Additionally, COVID‐19 patients exhibit a range of symptoms, from being asymptomatic to requiring intensive care and, ultimately, leading to death.^5^ Early prediction of the prognosis and severity of the disease during the diagnostic process is crucial for effective treatment and making appropriate medical decisions.^6^

The gold standard for diagnosing COVID‐19 is the molecular detection of the coronavirus genome using quantitative polymerase chain reaction (qPCR).^7^ The *N, E*, and *S* genes, *RNA‐dependent RNA polymerase* (*RdRp*), and *ORF1 a/b* genes are utilized to detect COVID‐19. Samples identified with these genes indicate that a person is infected with the SARS‐CoV‐2 virus or is suffering from COVID‐19 disease.^8^ However, it is important to note that mutations in the virus can lead to false‐negative results in the qPCR test.^9^, ^10^ Several studies have shown a correlation between the quantification cycle rate, viral load, and the severity of COVID‐19 disease.^11^, ^12^

In addition to qPCR, high‐risk populations are often recommended to undergo a complete blood count (CBC) and inflammatory tests concurrently. Checking the blood tests in carriers, symptomatic individuals, and those suspected of being infected with the COVID‐19 virus can be useful before performing the qPCR test.^13^ According to studies, leukopenia and lymphopenia are two characteristics commonly observed in COVID‐19 patients. Additionally, various studies have examined the diagnostic usefulness of nonspecific inflammatory biomarkers such as acute phase reactive protein (CRP), white blood cell count (WBC), and absolute neutrophil (Neu) count.^14^ Infection with SARS‐CoV‐2 can trigger a “cytokine storm,” which involves the excessive release of proinflammatory cytokines and can lead to acute lung injury and a poor prognosis.^15^ Therefore, assessing laboratory parameters associated with increased inflammation can aid in the diagnosis, prognosis, and treatment of COVID‐19.

CRP is a plasma protein induced by inflammatory mediators like interleukin‐6.^16^ Although it is a nonspecific marker, elevated CRP levels are used as an indicator of inflammation, and its elevation has been linked to disease severity.^17^ Another parameter commonly used in assessing inflammatory conditions is the erythrocyte sedimentation rate (ESR). Inflammatory conditions cause an increase in fibrinogen levels, leading to red blood cells sticking together and raising the ESR value.^18^ D‐dimer, on the other hand, refers to two fragments of the fibrin protein released into the bloodstream through the breakdown of blood clots by plasmin. It plays a significant role in the activation of cytokines in COVID‐19 disease.^19^ Furthermore, lactate dehydrogenase (LDH) is an enzyme that converts pyruvate to lactate and is released due to membrane damage in cells.^20^ Viral infections and lung injuries like pneumonia and COVID‐19 can cause LDH levels to increase, and this increase has been associated with the development of COVID‐19 disease.^21^

The aim of this study was to detect the coronavirus using qPCR and determine the correlation between the Cq level and the results of hematological and inflammatory tests. Additionally, the study sought to investigate whether the Cq level is a suitable parameter for providing information about COVID‐19 disease.

## 2 MATERIALS AND METHODS

This case–control study was conducted on 234 individuals referred to NIKAN Laboratory in Mashhad, Iran. Initially, nasopharyngeal and oropharyngeal samples were obtained, and a qPCR test was conducted on the study population. Based on the Cq level, the study population was divided into two groups: cases and controls. Samples with a Cq < 40, as determined by the Pishtaz Teb kit, were considered positive and categorized as the case group. Additionally, blood samples were collected for hematology, biochemistry, and inflammatory tests. Demographic characteristics were recorded, and laboratory tests including CRP, ESR, LDH, and D‐dimer, as well as CBC and IgG and IgM levels in the study population, were performed. The control group was selected by excluding patients with abnormal laboratory parameters from the study.

### 2.1 Hematological, immunological, and biochemical tests

The CBC test was performed using a calibrated cell counter (Sysmex XP 100). ESR was measured with an optical reader (Therma NE 100). D‐dimer levels were determined using the Chemiluminescence method (IMMULITE 2000 XPI). Quantitative C‐ reactive protein levels were measured turbidometrically, while LDH enzyme levels were determined using colorimetric methods, both with an autoanalyzer (OLYMPUS AU640).^22^ Additionally, COVID‐19 IgM/IgG rapid tests were conducted using the ELISA assay (Pishtaz Teb Kit).

### 2.2 Molecular test by qPCR method

RNA extraction was performed using the total RNA column isolation kit (BehGen). The quality and quantity of the extracted RNA were assessed using a Nanodrop spectrophotometer (Thermo Scientific) at *A*_260 nm_/*A*_280 nm_.^23^ Complementary DNA (cDNA) was synthesized via reverse transcription using an Addbio cDNA kit and the extracted RNA template.^24^ The qPCR for the target genes was carried out using the Roche LightCycler® 96 Real‐Time PCR Instrument. RNaseP was used as an internal control to evaluate the accuracy of sampling and RNA extraction. Specific probes were utilized to identify the RdRp and N sequences in the COVID‐19 genome during the qPCR test. Each PCR reaction mixture contained 10 μl TaqMan PCR Master Mix (Pishtaz Teb), 2 μL of the first‐strand cDNA, 0.4 μL of each primer and probe, and dH_2_O to reach a final volume of 20 μL. The amplification program consisted of an initial denaturation step at 95°C for 30 s, followed by 40 cycles, with each cycle including two steps: denaturation at 95°C for 5 s, and annealing and elongation at 60°C for 30 s. Calibration curves for each target gene were generated using serial dilutions, and the PCR efficiency was calculated to be between 98% and 99%. Negative controls were included in each run.^25^

### 2.3 Statistical analysis

All results were presented as the mean and standard deviation (SD). Statistical analysis was conducted using SPSS 22 software. The data were divided into two groups: the case group and the control group. The normality of data distribution was assessed using the Kolmogorov–Smirnov and Shapiro–Wilk statistical tests. For comparisons between groups, the Mann–Whitney test, independent *t*‐test, Wilcoxon's test, and *χ* ^2^ test were performed. Pearson's and Spearman's tests were employed to examine the correlations between parameters. Additionally, receiver‐operating characteristics (ROC) curves were generated to calculate the sensitivity and specificity of the parameters.

## 3 RESULTS

In the current study, a total of 232 participants were enrolled. Based on the results of the qPCR test, the participants were divided into two groups: the case group and the control group. Among the participants, 131 individuals tested positive for the qPCR test with Cq < 40, indicating the presence of the virus in the case group. The gender distribution in the case group was 45% male and 55% female. The average Cq values for the positive individuals were 21.61 ± 0.37 for the RdRP gene and 21.11 ± 0.38 for the N gene. The association between gender and the Cq level of the qPCR test was investigated among COVID‐19 cases, but no significant correlation was observed. Various parameters, including age, hematological, and biochemical parameters, were evaluated and compared between the case and control groups. The results are presented in Table 1 **.**

**Table 1:** The mean and standard deviation of parameters and the comparison of parameters between case and control groups.

Parameters	Control (mean ± SD)	Case (mean ± SD)	*p* Value
Age (year)	37.48 ± 1.82	42.8 ± 1.70	.067
Neutrophil (×10^3^/μL)	4733.07 ± 1805.845	5983.97 ± 1226.6	.001*
Lymphocyte (×10^3^/μL)	2601.29 ± 696.786	1362.06 ± 1076.4	.002*
Platelet (×10^3^/μL)	277 × 10^3^ ± 646 ×10^3^	192.587 × 10^3^ ± 53.763× 10^3^	.001*
PLR	114.59 ± 42.43	159.074 ± 58.65	.000*
NLR	1.94 ± 0.944	5.02 ± 2.00	.000*
D‐dimer (mg/L)	259.8 ± 103.59	495.82 ± 380.040	.000*
ESR (mm/h)	9.59 ± 3.13	15.82 ± 10.90	.000*
CRP (mg/L)	3.61 ± 4.91	17.46 ± 22.34	.001*
LDH (U/L)	247.37 ± 74.71	359.63 ± 102.50	.000*

Abbreviations: CRP, C‐reactive protein; ESR, erythrocyte sedimentation rate; LDH, lactate dehydrogenase; NLR, neutrophil‐to‐lymphocyte ratio; PLR, platelet‐to‐lymphocyte ratio.

*p < 0.05.

According to the findings, the average counts of Neu, PLR, NLR, D‐dimer levels, ESR, CRP, and LDH levels were significantly higher in COVID‐19‐positive cases compared to normal cases. Conversely, the counts of lymphocytes (Lym) and platelets (PLTs) in the case group were significantly lower than those in the control group. The average age of the participants included in the study was 42.8 ± 1.7 years. Additionally, while the ages of individuals in the case group tended to be higher than those in the control group, the difference was not statistically significant (*p* > .05).

### 3.1 Correlation between laboratory parameters in COVID‐19 patients

To investigate the relationship between hematological and inflammatory parameters in COVID‐19‐positive cases, Spearman's test was conducted. Table 2 illustrates the associations between these parameters. The correlation between Cq level and Lym count was found to be statistically significant and positive. Conversely, the correlation between Cq level and NLR level was statistically significant but negative. Additionally, there was a significant positive correlation between D‐dimer level and ESR, CRP, and NLR. On the other hand, PLT and Lym counts showed a significant negative correlation with PLR. Furthermore, the ESR level exhibited a significant positive correlation with age and CRP, while the PLT count displayed a significant negative correlation with both. Moreover, there was a significant positive correlation between CRP level and ESR, LDH, and NLR, as well as a significant negative correlation between PLT count and Lym count. Lastly, the LDH level demonstrated a significant positive correlation with age and NLR.

**Table 2:** The correlation between parameters.

	Age	ESR	CRP	LDH	PLT	Lym	Neu	NLR	PLR	D‐dimer
Cq	*r* = .078	r = −.562	r = −.069	r = −.37	*r*= .124	*r* = .214	r = −.14	r = −.208	r = −.05	r = −.138
	*p* = .379	*p* = .527	*p* = .433	*p* = .727	*p* = .160	*p* = .014*	*p* = .194	*p* = .017**	*p* = .537	*p* = .116
D‐dimer	r = .25	r = .20	r = .19	r = 0.11	r = −.72	r = −.201	r = .047	r = .166	r = −.395	
	*p* = .004*	*p* = .017*	*p* = .02*	*p* = .20	*p* = .00*	*p* = .021*	*p* = .59	*p* = .057*	*p* = .000*	
ESR	*r* = .417		r= .480	r= .137	r= −.113	r= −.231	*r* = −.59			r = .208
	*p* = .000*		*p* = .000*	*p* = .120	*p* = .267	*p* = .008*	*p* = .505			*p* = .017*
CRP	r = .163	r= .480		r= .198	r= −.174	r = −.239	r= .137	r= .251	r= .021	r= .199
	*p* = .062	*p* = .000*		*p* = .024*	*p* = .046*	*p* = .006*	*p* = .118	*p* = .004*	*p* = .814	*p* = .023*
LDH	r = .198	r = .137	r= .198		r= −.122	r= −.111	r= −.44	*r*= .045	r = .23	*r*= .112
	*p* = .024*	*p* = .120	*p* = .024*		*p* = .166	*p* = .298	*p* = .616	*p* = .606	*p* = .790	*p* = .204
PLT	r= −.374	r= −.113	r = −.174	r= −.122						r= −.72
	*p* = .000*	*p* = .267	*p* = .46	*p* = .166						*p* = .000*
Lym	r = −.102		r= −.239	r = −.111						r = .047
	*p* = .221		*p* = .006*	*p* = .298						*p* = .597
Neu	r= −.124	r = −.114	r = .137	r = −.44						r = .047
	*p* = .158	*p* = .194	*p* = .118	*p* = .616						*p* = .597
NLR	r= .020									
	*p* = .818									
PLR	r = −.2									
	*p* = .022*									
IgM	*r*= .160	r = .213	r= 0.068	r= .135	r= −.283	r = −.150	*r*= .07	r = .115	r= −.084	r = .266
	*p* = .453	*p* = .014*	*p* = .441	*p* = .123	*p* = .001	*p* = .087	*p* = .382	*p* = .190	*p* = .341	*p* = .002*
IgG	r= .118	r= .236	r = .067	r = .075	r= −.158	r = −.185	r= .17	r= .130	r = .029	r = .122
	*p* = .356	*p* = .007*	*p* = .449	*p* = .396	*p* = .071	*p* = .035	*p* = .847	*p* = .139	*p* = .739	*p* = .164

Abbreviations: CRP, C‐reactive protein; ESR, erythrocyte sedimentation rate; LDH, lactate dehydrogenase; Lym, lymphocyte; Neu, neutrophil; NLR, neutrophil‐to‐lymphocyte ratio; PLR, platelet‐to‐lymphocyte ratio; PLT, platelet.

*p < 0.05.

### 3.2 Evaluating hematological and inflammatory indices in COVID‐19 patients using ROC curve analysis

**Figure 1:**
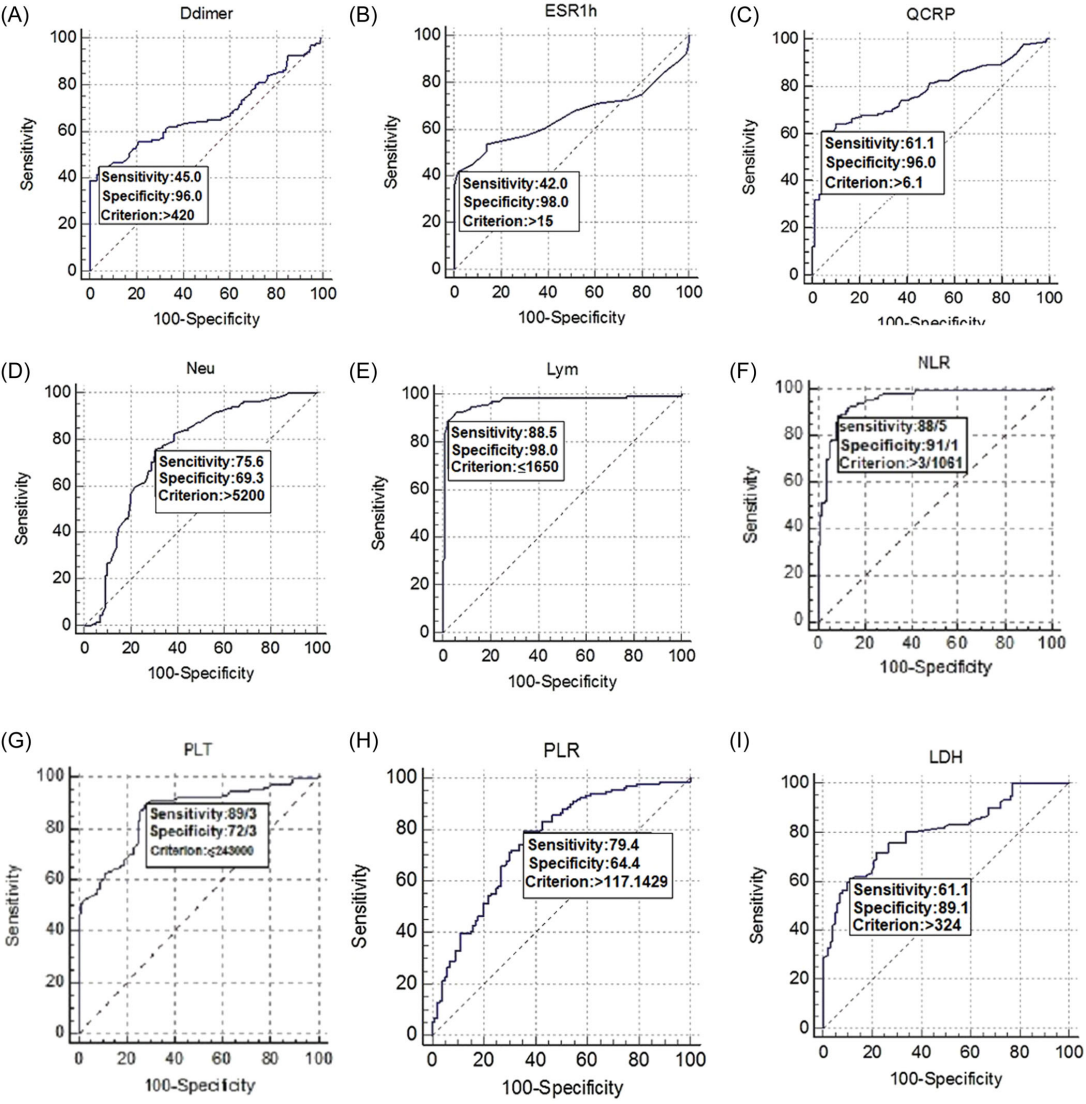
Evaluation of hematological and inflammatory indexes in coronavirus disease 2019 patients using the receiver‐operating characteristic curve. The parameters of D‐dimer (A), erythrocyte sedimentation rate (B), C‐reactive protein (C), neutrophil (D), lymphocyte (E), neutrophil‐to‐lymphocyte ratio (F), platelet counts (G), platelet‐to‐lymphocyte ratio (H), and lactate dehydrogenase (I) parameters showed statistically appropriate sensitivity and specificity, while the age was not significant statistically.

To assess the diagnostic significance of D‐dimer, ESR, CRP, LDH, Lym, Neu, PLTs, NLR, and PLR parameters, we conducted the ROC curve analysis based on the data obtained from both patients and control groups. The results of the ROC curve analysis can be found in Table 3 and Figure 1.

**Table 3:** ROC curve analysis of hematological, biochemical, and inflammatory parameters.

Parameters	Area under the ROC curve (AUC)	Significance level *p*	95% Confidence interval	Sensitivity	Specificity	Cut‐off
Age	0.570	0.0648	0.504–0.635	58	54.5	>36
D‐dimer	0.676	<0.0001	0.612–0.736	45	96	>429
ESR	0.656	<0.0001	0.591–0.717	42	98	>15
CRP	0.779	<0.0001	0.720–0.831	61.1	96	>6.1
LDH	0.802	<0.0001	0.745–0.851	89.1	61.1	>324
Lymphocyte	0.968	<0.0001	0.937–0.987	88.5	98	<1650 × 10^3^
Neutrophil	0.748	<0.0001	0.687–0.802	75.6	69.3	>5200 × 10^3^
Platelet	0.859	<0.0001	0.808–0.901	89.3	72.3	<243 × 10^3^
NLR	0.949	<0.0001	0.913–0.974	88.5	91.1	>3.10
PLR	0.756	<0.0001	0.695–0.810	79.4	64.4	>117.14

Abbreviations: CRP, C‐reactive protein; ESR, erythrocyte sedimentation rate; LDH, lactate dehydrogenase; NLR, neutrophil‐to‐lymphocyte ratio; PLR, platelet‐to‐lymphocyte ratio; ROC, receiver‐operating characteristic.

## 4 DISCUSSION

In the WHO guidelines for COVID‐19 diagnosis, the qPCR test was introduced as the gold standard. Several studies have shown a link between Cq and viral load and COVID‐19 disease severity. Cq levels in COVID‐19 patients are inversely proportional to virus load and disease severity.^26^ In this study, we examined the Cq level as an indicator of the COVID‐19 viral load and its correlation with various parameters, including gender, age, D‐dimer, ESR, CRP, CBC, NLR, PLR, LDH, IgG, and IgM levels. The minimum and maximum Cq levels for the RdRp and N genes in COVID‐19 were 12.14, 13.21, and 32.12, 33.18, respectively.

We found no significant correlation between age and Cq level, which aligns with the results of Ur Rehman et al.^26^ Mahallawi et al. in 2021 reported a range of 15.08–35, with an average of 5.23 ± 27.44 for Cq level, and there was no significant correlation between Cq level and gender.^27^ However, an earlier study in 2020 showed that men tended to experience more severe cases than women.^28^

WBCs, NLR, and PLR are indicators of systemic inflammatory responses and are widely used as prognostic markers for pneumonia.^29^ Compared to the normal group, COVID‐19 patients had significantly lower PLT and Lym counts, while Neu counts were significantly higher. Thrombocytopenia, lymphopenia, and neutrophilia can therefore be indicators of COVID‐19 disease, consistent with earlier studies.^11^, ^30^

According to current findings, lymphopenia, eosinopenia, and NLR > 3.13 were associated with greater COVID‐19 severity and poorer prognosis, while thrombocytopenia was associated with an increased risk of cardiovascular damage and poorer prognosis.^31^ Seddigh‐Shamsi et al. reported that COVID‐19 patients admitted to the intensive care unit had lower PLR levels and higher NLR levels were directly correlated with longer hospitalization.^32^

We also investigated the correlation between Cq level and CBC, NLR, and PLR. The correlation between Cq level and Lym count was significant and direct (*r* = .214), indicating that as the Cq level decreases, the Lym count decreases, and the NLR level increases. However, there was no significant correlation between Cq level and Neu and PLT counts, suggesting that the viral load of COVID‐19 patients does not affect these counts.

The Cq value can offer insights into the stage and severity of COVID‐19 infection. Studies indicate that patients with lower Cq values, corresponding to higher viral loads, tend to experience more severe infections or are in the early stages of the disease.^33^, ^34^ Our study suggests that the rate of SARS‐CoV‐2 infection may be significantly correlated with various laboratory parameters. Consequently, these indexes could potentially serve as indicators of disease severity, requiring further investigation.

In our study, we observed significantly higher levels of CRP in COVID‐19 cases compared to the control group. This elevation in CRP levels can be attributed to the production of CRP induced by inflammatory cytokines and tissue destruction, particularly in severe COVID‐19 patients.^35^ According to the area under the ROC curve (0.77), with a sensitivity rate of 61.1% and a specificity rate of 96%, CRP proved to be a suitable biomarker for diagnosing COVID‐19. Therefore, an increase in CRP levels can serve as a predictor for COVID‐19 prognosis and aid clinicians in early diagnosis. Our investigation also revealed a direct and significant association between CRP and other parameters such as ESR, LDH, NLR, and D‐dimer. This correlation can be attributed to the increased production of inflammatory cytokines in COVID‐19 patients. Iwamura et al. demonstrated that both CRP and ESR levels rise irrespective of the severity of COVID‐19 disease and underlying conditions. In our study, we observed significantly higher CRP levels in COVID‐19 cases compared to the control group, primarily due to the activation of CRP production caused by inflammatory cytokines and tissue damage, especially in severe COVID‐19 cases.^36^ Furthermore, we found a significant inverse correlation between CRP increment and Lym count decrease. Additionally, there was a direct and significant correlation between CRP and ESR levels. These findings can be attributed to the activation of the coagulation cascade, cytokine‐induced fibrin generation, and the presence of severe inflammatory conditions characteristic of COVID‐19.^37^, ^38^

Another parameter investigated in this study was D‐dimer, which has been reported to be higher in COVID‐19 patients who succumbed to the disease compared to those who recovered.^39^ Furthermore, Huang et al. showed that admission D‐dimer levels can predict the need for intensive care in COVID‐19 cases.^40^ In our study, the case group exhibited significantly higher D‐dimer levels than the control group. Abdulkareem and colleagues conducted a study that found moderate and direct associations between CRP and ESR levels with D‐dimer quantity, suggesting that an increase in these two inflammatory parameters can predict an elevation in D‐dimer levels.^18^ Our investigation also revealed a statistically significant inverse correlation (*p* = .000, *r* = −.72) between D‐dimer levels and PLT count. PLT count and D‐dimer concentration serve as strong indicators of thrombotic inflammation and COVID‐19 severity. It has been observed that as PLT count decreases, the levels of D‐dimer and mortality rate also change accordingly.^41^ Although we did not find a link between Cq and D‐dimer levels, elevated D‐dimer levels in COVID‐19 patients serve as accurate biomarkers for predicting disease severity and mortality.

LDH was another marker examined in this investigation, and our data showed significantly higher LDH levels in the case group compared to the control group, although there was no significant correlation between Cq and LDH. However, some studies have shown that decreased Cq levels are significantly correlated with elevated LDH levels.^42^

Assessing hematological, biochemical, and inflammatory markers along with their interactions, including the Cq level, was one of the strengths of this study. However, similar to other studies, limitations such as the lack of access to patients' clinical symptoms were present. On the other hand, the investigation of C4 and C3 components of the complement pathway holds significant potential, but due to limited financial resources, these experiments were not performed. Additionally, conducting this research with a larger sample size could provide more precise statistical data. Future research incorporating these assessments could provide valuable insights into the relationship between inflammation and COVID‐19.

## 5 CONCLUSION

In COVID‐19 patients, hematological inflammatory indicators such as CRP, ESR, and D‐dimer are elevated. Furthermore, a significant inverse relationship has been found between Cq values and Lym counts, along with a significant positive correlation with NLR values. Consequently, simultaneous reporting of CBC results, inflammatory parameters, and D‐dimer, alongside Cq values in qPCR tests, can aid clinical professionals and physicians in predicting the prognosis of COVID‐19 patients and making more informed decisions regarding their condition.

## AUTHOR CONTRIBUTIONS

**Hadi Safdari**: Investigation; validation; writing—original draft. **Saeede Bagheri**: Investigation; validation; writing—review and editing. **Nasrin Talkhi**: Data curation; formal analysis. **Elahe Saberi Teymourian**: Software. **Mahdi Hosseini Bafghi**: Data curation; project administration; supervision; validation. **Mohammad Hossein Ahmadi**: Data curation; project administration; supervision; validation. All the authors have read and approved the manuscript.

## CONFLICT OF INTEREST STATEMENT

The authors declare no conflict of interest.

## ETHICS STATEMENT

We prioritize human rights, welfare, and privacy. We obtain informed consent from participants, protect their anonymity and confidentiality, and minimize any potential harm or discomfort they may experience because of their involvement. We comply with all relevant laws, regulations, and guidelines governing research ethics. This includes obtaining necessary approvals from institutional review boards or ethics committees and adhering to applicable data protection and privacy regulations. This research has ethics approval with ID: IR.MUMS.FHMPM.REC.1401.119 issued by Mashhad University of Medical Sciences, Mashhad, Iran.

## Data Availability

The data that support the findings of this study are available from the corresponding author upon reasonable request.
